# ‘We are in this together’ voices of speech-language pathologists working in South African healthcare contexts during level 4 and level 5 lockdown of COVID-19

**DOI:** 10.4102/sajcd.v68i1.792

**Published:** 2021-02-18

**Authors:** Skye N. Adams, Jaishika Seedat, Kim Coutts, Kelly-Ann Kater

**Affiliations:** 1 Department of Speech Pathology and Audiology, School of Human and Community Development, Faculty of Humanities, University of the Witwatersrand, Johannesburg, South Africa

**Keywords:** COVID-19, Coronavirus disease, healthcare workers, service delivery, South Africa, speech-language pathologist

## Abstract

**Background:**

SARS-CoV-2 (COVID-19) has had a significant impact on every South African but more specifically healthcare professionals, including speech-language pathologists (SLPs). In response to the COVID-19 pandemic, South Africa implemented a nationwide lockdown as confirmed cases continued to rise. Understanding the impact of COVID-19 on SLPs has a three-fold purpose: to re-evaluate service provision, service delivery platforms and to identify the need for support to SLPs during a time of crisis. It is also crucial in guiding how policies and interventions need to be modified.

**Objectives:**

The study aimed to better understand how the workspace of SLPs in hospitals was impacted by COVID-19, how they experienced this process and the implications for them as healthcare professionals in both the private and public sector throughout South Africa.

**Methodology:**

An exploratory cross-sectional study design was used to meet the aims of the study. Thirty-nine SLPs from different provinces in South Africa, working in government and private hospitals during COVID-19, responded to the online survey. Results were analysed using descriptive statistics and thematic content analysis.

**Results:**

SLPs’ roles, responsibilities and service delivery were impacted by COVID-19. It was necessary for typical outpatient therapy services to be modified; there were changes to the role of the SLP in the hospital and inpatient services were curtailed.

**Conclusion:**

This study provides insightful information to SLPs employed in hospitals to know that they are experiencing similar challenges. It also confirms the resilience of healthcare professionals, including SLPs, when faced with novel and unprecedented situations.

## Introduction

The outbreak of the SARS-CoV-2 virus that causes coronavirus (COVID-19) in December 2019 caused a global pandemic within a short space of time (Lancet, 2020). Spreading from Wuhan, China, it quickly became necessary for countries across the world to institute different levels of lockdown to decrease the number of infections. As a result, South Africa (SA) entered a nationwide ‘hard’ level 5 lockdown on the 27 March 2020. The country came to an abrupt halt, as movement of all but essential service providers were restricted. Businesses closed and many, where possible, had to work from home. Despite the sanctions to limit and stop the spread of the virus, the number of infections continued to rise and hospitals became increasingly overwhelmed because of restricted resources. South Africa saw its peak in cases in July with approximately 1300 new cases being reported daily. Consequently, all healthcare professionals, including speech-language pathologists (SLPs), were impacted in various ways (Adams & Wall, 2020). The need arose to understand the impact that COVID-19 had and is continuing to have on patients, the health system, as well as on the different healthcare professionals. Furthermore, given the uncertainty around the development, acceptance and roll-out of a vaccine for COVID-19, the country needs to ensure that the impact on future health service provision is minimised. Therefore, SA will need to find ways to create surge capacity to treat COVID-19 patients whilst maintaining essential services. In some ways, COVID-19 may have levelled the playing fields in private and public healthcare with regard to access and resource allocation of healthcare services (Labuschaigne, 2020). In addition, South Africa’s healthcare provision in private and public is rife with inequalities with regard to who accesses these services and the availability of resources (Pillay, Tiwari, Kathard, & Chikte, 2020; Shai & Ogunnubi, 2018). Thus, it was important to understand how healthcare professionals in both private and public, who are working in low- and middle-income countries such as SA, experienced the impact of COVID-19 at the strictest levels of lockdown.

During COVID-19, as hospitals struggled with high patient loads, many healthcare professionals took on additional roles, including SLPs, which had an impact on their scope of work as well as service delivery. In addition, there has been an increase in the adverse effects of healthcare workers that include risk and exposure to COVID-19 (Cipolotti et al., 2020). Speech-language therapy professional boards worldwide published revised practice guidelines for the SLP during COVID (American Speech-Language-Hearing Association [ASHA], 2020; New Zealand Speech-language Therapists’ Association, 2020; Royal College of Speech and Language Therapists [RCSLT], 2020; Speech Pathology Australia, 2020). Governments globally initiated a clear directive to refocus service provision to emergency and high-risk, high-priority (in)-patients only (predominantly dysphagia and some post-surgical), with outpatient services either being postponed or provided via teletherapy (Mann, Chen, Chunara, Testa, & Nov, 2020). At the time the study was conducted (June 2020), an aspect that appeared to have been neglected somewhat was the impact of the change in roles, additional roles and the anxiety associated with potential exposure to COVID-19 on the mental and emotional well-being of SLPs. The current study sought to examine how SLPs working in healthcare facilities in SA experienced COVID-19, both professionally and personally.

### Speech language pathology and COVID-19

Hospital-based SLPs have had to adjust to the new policies and guidelines from the government regarding how to reconsider and revise their workspace and service provision to contain the infection and protect the most vulnerable amongst their patients. With hospitals redirecting admissions and making provisions for patients who require admission for COVID-19, there were a number of workload implications for SLPs as hospitals attempted to accommodate priority patients. Implications for a reduction in services to both inpatients and outpatients were thus inevitable (ASHA, 2020; Gregory, Henley, & Amaya, 2020; RCSLT, 2020). Coto, Restrepo, Cejas and Prentiss (2020) in their study on the impact of COVID-19 on allied health professionals (AHPs) reported that 10% of the 920 participants in their study were reassigned to perform other duties that included screening patients (2.1%), taking temperatures (2.1%), scheduling (1.6%) or triaging patients (1.1%). In SA, there was a need for all healthcare professionals to adhere to government regulations (South Africa Disaster Management Act No. 57 of 2002), for only essential services to be rendered and for staff to be re-deployed to roles and areas where health services were required within an institution. For SLPs (and other AHPs) it is unclear how hospitals defined essential and high-risk patients. Given the relationship between COVID-19 and respiratory compromise, these patients were predominantly those requiring dysphagia-related intervention. The SASLHA dysphagia guidelines provided some direction in this regard (SASLHA Dysphagia Guideline, 2020). At the time of the study, there was limited evidence on how general SLP services have been impacted and what this might mean for patient care as well as the impact on SLPs themselves who may be at risk for exposure to COVID-19. Thus, it was imperative to understand the implications COVID-19 may have had on the SLPs’ scope of practice, patient populations and service delivery both during this time and for the future.

Amongst the various areas that SLPs work in, dysphagia- and dysphagia-specific populations such as patients with tracheostomy and laryngectomy may pose an increased risk to SLPs because of their probability of coughing and need for suctioning (SASLHA Dysphagia Guideline, 2020). Both coughing and suctioning have been identified as aerosol generating procedures (Bolton, Mills, Wallace, Brady, & Group, 2020). Thus, the use of personal protective equipment (PPE), implementing social distancing and patient positioning are considerations that impact service delivery from the perspective of both the patient and the SLP. As SARS-CoV-2 has been identified in the saliva of infected patients, transmission through contact and respiratory droplets brought on by coughing and choking may be expected (Bolton et al., 2020; Sabino-Silva, Jardim, & Siqueira, 2020). Thus, there was a need to understand how SLPs working with patients and aerosol generating procedures cope, manage and provide these services. SLPs may have had to take on other responsibilities in the hospital such as COVID-19 screening over and above service delivery to high-risk patients. As hospital policies changed as levels of lockdown eased, knowledge of how this impacted the service provision by SLPs was important to explore, given the uncertainty of when repercussions of the pandemic will be resolved.

## Method

The objectives of the current study were as follows:

- to describe the impact that COVID-19 had on SLPs’ perceptions of working in healthcare contexts both on a personal and a professional level
- to understand how COVID-19 impacted service delivery specifically.

### Research design

An exploratory qualitative cross-sectional study design was used using an online questionnaire, based on the literature related to healthcare worker’s experiences working during COVID-19 (see Chew et al., 2020; Dimer et al., 2020; Franza et al., 2020) (Appendix 1). This design enabled the researcher to obtain an understanding of how SLPs were experiencing COVID-19 within the hospitals being studied (Creswell, 2014). In addition, based on the restrictions because of COVID-19, an online questionnaire was deemed most appropriate as it complied with the lockdown regulations of appropriate social distancing, was cost-effective and allowed for data to be collected in a short amount of time across different provinces.

### Participants

Participants were SLPs from across SA to represent a heterogenous group, diverse in their contexts and working conditions. Data were collected using an online questionnaire from participants who met the following inclusion criteria: (1) SLPs registered with the Health Professions Council of South Africa (HPCSA), (2) SLPs working at a public or private hospital and (3) SLPs working during the COVID-19 period.

Thirty-nine SLPs comprised the sample. Table 1 provides an overview of the provinces they worked in and their work setting. Most respondents were from Gauteng (*n* = 16), followed by Western Cape (*n* = 11), with 23 SLPs from the public sector, and 16 working in the private health sector, namely, 13 working in a private hospital and three SLPs working in a private rehabilitation context. It was unfortunate that the response rate was not higher, given that there are currently 2643 SLPs registered with the HPCSA (Pillay et al., 2020). Whilst acknowledged under the limitations, the researchers were cognisant of the timing of data collection – there were an unusually high number of surveys being completed at the time of the data collection because of the coronavirus pandemic. Thus, the sample of 39 was deemed acceptable. Furthermore, the data added value to how SLPs in healthcare settings were experiencing the pandemic in the South African context.

**TABLE 1:** Participant description and characteristics.

Parent characteristics	*N*	%
**Age (years)**		
18–29	22	56.4
30–39	9	23
40–49	5	12.9
50–59	3	7.7
**Province**		
Gauteng	16	41.2
Eastern Cape	3	7.7
Kwazulu-Natal	2	5.1
Western Cape	11	28.2
Limpopo	3	7.7
Mpumalanga	2	5.1
Free State	2	5.1
**Type of hospital/clinic**		
Private hospital	13	33.3
Private rehab hospital	3	7.7
State hospital tertiary	10	25.6
State hospital regional	8	20.7
District hospital	2	5.1
Rural hospital	2	5.1
Community clinic	1	2.6

### Procedures and data collection

Following ethics approval, the South African Speech Language and Hearing Association (SASLHA) was used to recruit participants. The web questionnaire for this study was conducted from 15 June 2020 to 29 June 2020 level 3 of the nationwide lockdown. All participants were provided with consent forms prior to completion of the online questionnaire. There was no risk of harm to the participants. Participants were also informed of their right to withdraw at any point in the study. The questionnaire consisted of both structured and open-ended questions, including:

- demographic information (understand where participants are from and their work setting)
- previous and current role/duties (work routines prior to and under COVID-19)
- challenges and benefits of working in the hospital as an SLP in the current context (facilitators and barriers of working under COVID-19 to better inform clinical practice for as long as COVID-19 pandemic is present)
- the impact of COVID-19 on their patients/services (to gain insights into how SLP prioritised patients and the reasons underlying their choices).

### Reliability and validity

Four experts who had experience in working in the field of SLP in the hospital setting reviewed the questionnaire. After review, all experts commented on the relevancy and appropriacy of the questionnaire, and no changes were made. This improved the face validity of the questionnaire. Following the review, the questionnaire was piloted and reviewed for validity in two participants.

### Data analysis

Descriptive statistics (i.e. frequencies) were performed using SPSS Version 22. Inductive and deductive thematic content analysis of responses to open-ended questions was used for additional context.

### Ethical consideration

This study was approved by the University of Witwatersrand, Human Research Ethics Internal Committee (non-medical) H20/05/01.

## Results

The results are presented in line with the stated objectives of the study.

### Objective 1: How did COVID-19 impact both a personal and a professional level?

Of the total sample, 32 SLPs identified that COVID-19 had impacted their typical role/s within their work setting and seven SLPs noted no change. In addition, of those who reported an impact, 23 were from the public sector. The major themes that emerged with regard to the impact of COVID-19 on SLP role within the work setting were as follows.

#### Theme 1: Changes to inpatient services

Service provision to individuals receiving intervention across the private–public spectrum was adversely impacted. SLPs reported that outpatient services stopped, and inpatient services were impacted by COVID-19 in different ways with some not seeing any patients as reported by P30 ‘… not yet seeing patients …’ and P29 ‘Currently none at all’, to not seeing patients under investigation (PUI) for COVID-19, ‘Currently not working with PUI or positive patients’ (P19), to only seeing patients when their results for COVID-19 came back negative, ‘… only see patients when test negative for COVID-19’ (P20), as well as continuing to see inpatients in the wards ‘I see patients in wards where there are persons under investigation for COVID-19’ (P2).

Changes to inpatient services were implemented to reduce the risk of infection for SLPs. However, P5 noted that whilst she is not in contact with COVID-19 patients she still risks infection from other hospital staff, ‘I am not involved with patients who have contracted COVID, however I am in contact with colleagues who may have had exposure to undiagnosed patients’ (P5). Thus, the exposure and transfer of the virus could not be circumvented completely given the nature of hospital work.

#### Theme 2: Additional roles taken on by speech-language pathologists during COVID-19

Ten SLPs noted being involved in a range of COVID-19 screening, prevention and promotion services and roles within the hospital that they would otherwise not have been required to fulfil:

‘I was part of the community screening and testing.’ (P2, Western Cape, Public sector)

‘We are currently helping out with community testing and screening …’ (P3, Western Cape, Public sector)

‘I participate in screening at the main entrance of the hospital in order to identify patients at risk who need to be triaged and tested for COVID-19.’ (P19, Gauteng, Public sector)

As noted above, despite precautions being taken to prevent transmission of infection by patients to SLPs, their involvement in hospital services, while noteworthy and necessary, may have inadvertently placed them at risk given the nature of screening, as noted by P19. This was also picked up in the response by P37 ‘I coordinate and perform screening of all outpatients and visitors to the hospital for COVID-19’. A theme that emerged in terms of the personal impact that SLPs experienced from COVID-19 that of *Vulnerability* and *Risk (see below).* Results suggested that the personal risk impact was underpinned by the additional roles that SLPs were involved in.

In addition to screening, other additional roles taken on by SLPs included health prevention and promotion regarding COVID-19 such as community education:

‘We do health promotion and education in the community regarding COVID-19 signs and symptoms as well as infection control (i.e. washing hands, wearing a mask, etc.)’, (P3, Western Cape, Public sector)

As well as running tutorials and compiling policies around infection control and sanitisation:

‘… I am a member of the COVID Crisis Task Team at the hospital. I am involved in writing and proof-reading SOPs and policies at the hospital, which are around COVID-related issues currently’. (P37, Western Cape, Public sector)

**Personal impact of COVID-19: Vulnerability and risk:** Twenty-nine of the 39 SLPs noted that they felt comfortable in their personal knowledge about COVID-19. Thus, whilst they were aware of what COVID-19 was, how it was spread, protocols that needed to be adhered to and they had knowledge of infection control procedures, feelings of risk were still expressed by all participants on a scale from feeling at high-risk to low-risk. Some reasons underpinning this anxiety as expressed by participants were the need for more factual data by their employers, regular statistics, updates on the virus itself in terms of spread and reinfection, as well as more attuned training and information for AHPs:

‘More transparency on numbers of infected patients, where in the hospital they are situated, deaths, etc.’. (P1, Eastern Cape, Public sector)

‘It would be helpful to know the most up-to-date information, with supportive evidence, on certain aspects such as safety protocols to protect both patients and staff, current symptoms being experienced by the South African population, and when a person with COVID-19 is most infectious versus when it is safe to reintegrate into a normal ward’. (P4, Western Cape, Private sector)

‘Information about contracting the virus a second time? What are known expectations around that? Is it worse/better the second time? What’s the expected time frame?’ (P6, Western Cape, Private sector)

‘I would like more guidelines from hospital management on how to manage isolation and quarantining if in contact with PUI’s or Confirmed positives without adequate PPE’. (P20, KwaZulu Natal, Private sector)

Thus, a need for not only tangible resources in the way of PPE was necessary to reduce feelings of risk, but reassurance in the form of information was needed to enable SLPs to feel protected and considered by their employers. SLPs commented that attention to these aspects by employers would assist with the emotional and psychological distress they were experiencing, P22 ‘Hospital does not provide support regarding mental health’. One participant specifically noted that information provision, coupled with attention to psychological well-being for their employees, would cultivate feelings of trust by the healthcare workers:

‘Management needs to communicate the status of the hospital with respect to the number of staff who are infected or are PUIs, as well as the number of patients admitted with COVID, to prevent rumours and create trust. There is a need for information about the process of contact tracing. Management needs to set clear guidelines for the wearing of appropriate PPE and take steps to prevent wastage and ensure consistent availability of PPEs’. (P37, Western Cape, Public sector)

#### Theme 3: Improved inter- and intra-professional teamwork

Twenty-five of the 32 participants, who noted that there was an impact on them professionally, felt that the impact was positive, with improvement in teamwork emerging as the major theme both intra- and inter-professionally:

‘Better team work with Nurses, Doctors and the rest of the multidisciplinary team, a feeling of “**We’re all in this together**”’. (P27, Western Cape, Private sector)

‘We are working together as a team with community based care workers, nurses and allied health team’. (P3, Western Cape, Public sector)

‘Increased team work in department and MDT’. (P23, Gauteng, Public sector)

‘We now collaborate with pharmacy as well and doctors recognise our scope more. We are becoming involved in cases that typically we would be overlooked for’. (P35, Gauteng, Prublic sector)

Therefore, despite the changes COVID-19 had on the SLPs’ role in the hospital, many still reported a positive impact, specifically around teamwork both within the department and with other multidisciplinary members. This highlighted the notion of ‘being in this together’ and the sense of community that the lockdown ‘enforced’. This was described in more detail:

‘My department has shown their solidarity and support towards each other and their commitment to their patients. We have tried new ways of engaging with our patients therapeutically, which will broaden the way we serve our patients after this pandemic is over. The crisis has allowed the staff with really strong work ethic and a heightened sense of commitment to the greater good of our patients to find each other and work together outside of the usual silos; there has been better collaboration and an opportunity to meet people from other departments and work on activities other than direct patient management, and this bodes well for the post-COVID hospital, if management is willing to use the strengths that the pandemic has revealed’. (P37, Western Cape, Public sector)

#### Theme 4: Increased time for departmental duties

In addition to lockdown improving healthcare staff collaboration and teamwork, it also provided the participants in this study the time and space to concentrate on departmental needs. As patient load reduced, it provided SLPs’ additional time to focus on aspects of their work that they did not always have time to do as stated:

‘Lots more time doing research, reviewing protocols … in order to improve services. We’ve also started projects like toy making from waste products that will be used to improve our relevance to context when we bring out patients back and also to show parents at home how to make toys to use in lockdown from waste’. (P7, Gauteng, Public sector)

‘Reviewing of our infection control protocols … Extensive work on our practice guidelines … Resource development … Emotional resilience and adaptability … Creating visibility about our services’. (P9, Gauteng, Private sector)

‘Time to reflect more on clinical experiences. Time to deep clean and re-arrange folders’. (P33, Western Cape, Private sector)

This was relevant for departments with fewer SLPs, where the typical work situation prioritised inpatient and outpatient services over other administrative tasks.

#### Objective 2: How did COVID-19 impact speech-language pathologist service delivery?

Only two participants noted that COVID-19 did not impact service provision to patients, with 37 noting a significant impact. The two participants were from the private sector. All participants working in the public sector agreed on the impact of COVID-19 on service provision for patients.

**Theme 1: Quick discharge and COVID-19 inpatient prioritisation:** With regard to inpatients, SLPs reported that COVID-19 patients were prioritised which resulted in discharging patients very quickly as reported:

‘Patients are being discharged very quickly, limiting our provision of services in the acute phase’. (P19, Gauteng, Public sector)

‘Patients from referring hospitals are being discharged from hospital at a rate that we cannot match’. (P4, Western Cape, Private sector)

However, SLPs did not find this ideal and felt that it compromised patient prognosis, and potentially necessary assessment and intervention, as they sometimes did not get to see the patient prior to discharge:

‘Non-COVID patients are discharged very quickly, often within 24 hours. Little opportunity for follow-up because of lockdown restrictions’. (P27, Western Cape, Private sector)

‘Patients are admitted and then discharged very quickly without adequate time for full assessment or treatment’. (P32, Gauteng, Private sector)

P6 noted, that when seen, it was a time-consuming process:

‘Seeing COVID-19 patients is much more time consuming given all the donning and doffing…’. (P6, Western Cape, Private sector)

‘Sessions have been cut shorter because of limiting the exposure time to patients. Masks have affected the way in which OSME and OME are done as many patients have difficulty with understanding the STA’. (P20, KwaZulu-Natal, Private sector)

‘Inpatient hospital numbers have reduced significantly – doctors discharging patients home straight from the acute setting, patients and families do not want to be in a hospital setting with no visitors allowed. The expense of PPE, not being able to move between units if treated a PUI …’. (P31, Western Cape, Private sector)

As can be seen from these excerpts, a variety of factors and considerations were impacting inpatient services under COVID-19 and with good reason.

**Theme 2: Decreasing and stopping of outpatient services:** The outpatient populations who were most impacted were paediatrics (noted by nine participants), and generally high-risk patients (noted by all 35 participants). These included patients with comorbidities, immunocompromised patients and high-risk patients (tracheotomised patients):

‘Children who are high risk to come to a hospital and be exposed (children with Down syndrome & Trachy). Adults who have just suffered a stroke or TBI’. (P20, KwaZulu-Natal, Private sector)

‘CP patients (immunosupressed patients)’. (P16, Eastern Cape, Public sector)

‘Those fall into high-risk groups such as the elderly or those with co-morbidities’. (P12, Mpumalanga, Public sector)

Eleven participants out of 37 commented that services to outpatients had stopped:

‘Not allowed to see any speech therapy out-patient’. (P10, Limpopo, Public sector)

‘We have had to stop our outpatient clinics’. (P22, Eastern Cape, Public sector)

It was, however, noted that changes to outpatient services impacted and improved inpatient services:

‘Outpatients are not being seen although inpatient care has probably improved’. (P1, Eastern Cape, Public sector)

**Theme 3: COVID-19 protocol adversely impacting patient care:** Twenty-nine participants reported that their hospitals had specific COVID-19 PPE protocols in place, although there was a lack of PPE at certain hospitals:

‘Some private hospitals not providing appropriate PPE, therefore pt [*patient*] was unable to be seen immediately’. (P17, Western Cape, Private sector)

It is for this reason that the practice of donning and doffing, use of masks, social distancing and disinfection protocols were crucial regardless of the challenges this posed for effective service delivery:

‘It has impacted on the manner in which I interact with my patients. For communication it has resulted in significant difficulties with the facilitation of treatment for patients with cognitive and speech deficits who rely on visual stimulus (i.e. seeing your mouth) to participant [participate] in communication/therapy. It has been difficult to provide them with the best care …’. (P26, Gauteng, Private sector)

P26 states the negative impact the mask had on patient interactions in that patients with cognitive and linguistic challenges could no longer benefit from the use of visual cues.

**Theme 4: Redirection of speech-language pathologists services:** For outpatients, patients were redirected to receive therapy using a hybrid model which composed of telephonic consultations, teletherapy via a device, home programmes, caregiver training or WhatsApp as described by participants:

‘Telephone calls to follow up. WhatsApp video calls. Teletherapy for a very small percentage’. (P14, Western Cape, Private sector)

‘Tele rehab for those who are able to. We are also keeping strict lists of patients who aren’t receiving any therapy now (if they non urgent or and can’t do tele rehab) in order to prioritize at in person appointments them when it is safe to do so’. (P6, Western Cape, Private sector)

‘Offer of home programmes and caregiver training. Follow-ups and referrals for families who did not wish to continue virtual therapy’. (P32, Gauteng, Private sector)

However, in instances where the outpatient was unable to accommodate this, therapy stopped. Patients, who were required to travel to hospitals for services, were considered high-risk, and so discouraged from attending therapy and rather were given the option of a tele-form of service delivery (noted by 12 participants), failing which no services were provided. Patients, who were not comfortable coming into a hospital, were also offered a tele-form of service, and again, if they were unable to accommodate this, then they too received no service.

## Discussion

The data from this study provided valuable insights into the workspace of SLPs in hospital settings, who worked during level 4 and 5 of lockdown in SA. Not only were there implications for SLPs themselves, but for the patients, and other health and medical professionals, as well as management structures and perhaps professional bodies and training institutions. Current literature abounds with information around the management of COVID-19 patients and those presenting with respiratory concerns, breathing challenges, as well as patients with comorbidities especially diabetes and cardiac conditions (see Adams & Wall, 2020; Cipolotti et al., 2020; Koliaki et al., 2020; Sommer et al., 2020). These studies, many of which are medically oriented, are published almost daily, providing valuable direction for the management of COVID-19 patients. However, a concern raised in the current study was that there were a number of patient population groups who may have been neglected during this COVID-19 era. The medically stable patients, who still require some form of rehabilitation for improved functioning and prognosis, are often not receiving optimal and timely services. These patients include majority paediatrics as well as those as being labelled as high risk (i.e. patients presenting with comorbidities). As a result, these patients are placed on waiting lists and only being seen later creating a patient backlog.

The literature with evidence that talks to the importance of acute phase and regular and consistent intervention (Bicas, Guijo, & Delgado-Pinheiro, 2017; Gravel et al., 2020; Houtrow & Murphy, 2019) has had to be ignored to focus and manage the current situation. However, given the context of the South African healthcare system with regard to the already backlogged waiting lists and limited availability of therapists, this creates a significant problem. COVID-19 brought along its own stressors and anxieties for healthcare workers (Coto et al., 2020), but healthcare and health service provision in the post-COVID arena is something that will require creative input and possible collaboration from various stakeholders in different sectors for this already struggling sector in SA to withstand the onslaught from this pandemic. The results of the current study suggest that these negotiations should already be in progress as current lockdown restrictions have been eased, as participants commented on already excessive waiting lists.

Another aspect raised in the study was how the regulations such as social distancing, mask use and use of gloves were barriers in therapy situations, for both inpatients and outpatients. Given the nature of the work as SLPs, these barriers, while required, place SLPs (and likely other healthcare workers) in a juxtaposition. This situation forces SLPs to assess whether the services that are being offered are effective, valuable and whether those who require services are being seen. Furthermore, given the shortened duration of interactions, and the inconsistent basis on which patients are seen, which is contrary to the evidence (Bicas et al., 2017; Gravel et al., 2020; Houtrow & Murphy, 2019), it is difficult to justify certain service delivery decisions under COVID-19 restrictions. The current study thus raises many questions around delivery of patient services (or lack thereof), and Andrews et al. (2020) in their paper also grappled with aspects around patient triage and prioritisation, as well as rationing of services to cater for those with COVID-19. They further deliberated perceptions about quality of life and social determinants of health that resonate with the perceptions by some of the participants in the current study.

The current study did address some positive changes noted by SLPs, which was the aspect of improved inter- and intra-professional teamwork. These results are supported in the current literature with regard to how an increase in how teamwork, sense of solidarity and internal support amongst healthcare workers was present (Liu et al., 2020; Salas-Vallina, Ferrer-Franco, & Herrera, 2020). SLPs stated that although COVID-19 resulted in a number of negative experiences and changes in their current roles, there was still an internal resilience and spirit of togetherness amongst SLPs and their colleagues. However, it must be noted that participants in the study spoke to the need for additional support from hospital management, specifically in terms of being provided with the minimum resources to ensure that the work could be done efficiently, effectively and safely. This included the availability of PPE, being kept updated with information on COVID-19 as well as training on infection control protocols.

Another point of success that emerged from this study was the improved attention to infection control and the diligence with which infection control and sanitisation practices were implemented over the COVID-19 period. Similar trends were noted in studies by Barba et al. (2020) and Shanafelt, Ripp and Trockel (2020) that documented the benefits of improved adherence to infection control protocols. In line with the improved teamwork that materialised as discussed above, the responses from the participants belied a sense of ownership for their place of employment. When analysed across participants, it was clear that many participants saw themselves as healthcare professionals first. Their flexibility to take on roles that were not typical such as screening, prevention and promotion tasks to patients and communities alike, compiling policies and protocols, suggested that they were able to work outside of the typical SLP role. Acknowledgement for SLPs to be equipped to embrace these kinds of roles seamlessly must, in part, be attributed to the minimal exit level outcomes stipulated for undergraduates by the HPCSA (Annexure K: Regulations Relating to the Undergraduate Curricula of Speech Language Pathology). The importance for SLPs not only to be trained on content-specific material, but to have a broad overview in terms of collaboration, management, information provision, coordination and compiling protocols, as part of their training, to ensure that they had the skills to carry through with tasks that required these skills when practising, was evident from this study, and is something that the HPCSA advocates for.

Perhaps one of the most documented and published areas for many healthcare workers (as well as educators) currently is that of the implementation and benefits of tele-health services (Bustamante, Stevens, & Robinson, 2020; Law, Polovoy, Kornak, & Hutchins, 2020; Roger & Grimsley, 2020; Weidner & Lowman, 2020). The evidence clearly confirms that tele-health which has increased in use since COVID-19 can have remarkable benefits to many patients. Whist this was seen, to some extent, as reported by some participants, there was also evidence to the contrary for the South African context. This situation clearly highlighted the obvious technology gap that exists in our society, and this had a significant impact on service provision for many SLPs (Dimer et al., 2020; Pillay et al., 2020; Shai & Ogunnubi, 2018). Telehealth services, and the myriad forms this could take, was not an option for many patients, leaving SLPs with no alternative but to place them on waiting lists. With reference to the point made earlier regarding the ethics around this, it raises an awareness around how this may contradict what we learn about early and regular interventions. Thus, whilst feelings of anxiety about this were expressed from the participants in this study, perhaps a more necessary source of information, would be to gather the insights from the patients and caregivers themselves, on how this pandemic impacted them. This information then needs to be disseminated to higher bodies to accommodate the provision of these services. With the implementation of the National Health Insurance (NHI), teletherapy is definitely a mode that needs to be included and addressed.

The participants in the study expressed degrees of vulnerability, anxiety and perceptions of risk. Inadequate and irregular information, as well as poor distribution of information, were the main contributors to these feelings. Consequent implications for emotional and psychological well-being were highlighted in the current study. An increase in mental health conditions has spiked for a number of HCP during this time (Greenberg, 2020). This trend is not typical only to the South African context but was in fact seen globally and in all healthcare professions (Adams & Wall, 2020; Cipolotti et al., 2020; Pfefferbaum & North, 2020). This pandemic has further highlighted existing gaps in addressing and supporting the mental health and well-being of all healthcare workers. This is an area that also requires extensive collaboration and a unique solution.

Finally, the researchers considered if the COVID-19 experience by an SLP working in the public versus the private sector would be different. The results of this study did not confirm this. Whilst there appeared to be some differences in terms of how patient services changed, and how the role/s of the SLP also transitioned, it was not noteworthy. Although there were differences with regard to the management of in- and outpatients, SLPs generally shared similar experience with regard to the shortage of PPE and the impact of COVID-19 protocols on patient care and service delivery.

## Conclusion

This study provided valuable insight into the current experiences of SLPs, specifically during the COVID-19 era. These insights can assist various healthcare and academic institutions to address gaps such as telehealth practices, use of PPE in therapy and mental health challenges in future policies and guidelines. Positive factors such as improved team work and time for administrative tasks are perhaps aspects that need to be maintained going forward.

The authors are aware of a number of limitations of the current study. There was a small sample size in the current study relative to the number of practicing SLPs currently working in SA. The limited sample size could have been attributed to the number of surveys being sent to SLPs during this time and increased workload stressors. In the study, there was no quantitative measure of burden for the SLPs; in addition, using a self-administered form did not allow for the researcher to probe on certain topics. However, the researchers wanted to access participants during the pandemic in different geographical locations and use a method that was both convenient and cost-effective, thus the use of a questionnaire was deemed appropriate. Future research should include more interviews to get a more holistic picture of the challenges experienced by SLPs and other healthcare workers who are working in hospitals as well as other facilities (e.g. schools, private practice) during COVID-19.

## Clinical impact

The findings provided insightful information to the SLPs employed in both government and private hospitals, whereby they may be able to share experiences, problem-solve challenges and learn from each other. The results also provided vital information to guide student training once lockdown measures are eased. It will enable academics to prepare students for what they may encounter in the hospital and equip them with skills and resources to guide them in their experience upon return to the hospital. The findings also reveal caveats of excellence in professional training to equip graduates to be dynamic, think on their feet and be critical problem-solvers in unexpected and novel circumstances.
